# An efficient algorithm for data transmission certainty in IIoT sensing network: A priority-based approach

**DOI:** 10.1371/journal.pone.0305092

**Published:** 2024-07-17

**Authors:** Kemal Gökhan Nalbant, Sultan Almutairi, Asma Hassan Alshehri, Hayle Kemal, Suliman A. Alsuhibany, Bong Jun Choi

**Affiliations:** 1 Department of Software Engineering, Faculty of Engineering Architecture, Istanbul Beykent University, Istanbul, Turkey; 2 Department of Natural and Applied Sciences, Community College, Majmaah University, Al-Majmaah, Saudi Arabia; 3 Department of Computer Sciences, College of Computer Engineering and Sciences, Prince Sattam bin Abdulaziz University, Al Kharj, Saudi Arabia; 4 Faculty of Natural and Computational Science, Debre Berhan University, Debre Berhan, Ethiopia; 5 Islamic University Centre for Scientific Research, The Islamic University, Najaf, Iraq; 6 Department of Computer Science, College of Computer, Qassim University, Buraydah, Saudi Arabia; 7 School of Computer Science and Engineering, Soongsil University, Seoul, South Korea; New York University Abu Dhabi, UNITED ARAB EMIRATES

## Abstract

This paper proposes a novel cache replacement technique based on the notion of combining periodic popularity prediction with size caching. The popularity, size, and time updates characteristics are used to calculate the value of each cache item. When it comes to content replacement, the information with the least value is first eliminated. Simulation results show that the proposed method outperforms the current algorithms in terms of cache hit rate and delay. The hit rate of the proposed scheme is 15.3% higher than GDS, 17.3% higher than MPC, 20.1% higher than LRU, 22.3% higher than FIFO, and 24.8% higher than LFU when 350 different categories of information are present. In real-world industrial applications such as including supply chain management, smart manufacturing, automation energy optimization, intelligent logistics transportation, and e-healthcare applications, it offers a foundation for the selection of caching algorithms.

## 1 Introduction

With the rapid advancement of Internet of Things (IoT) technology, industrial automation systems are increasingly incorporating intelligent sensors and actuators with computational capacity, resulting in large volumes of IoT data [1]. This data can be used to improve the control process and optimize the production, and then increase the production efficiency. For example, control strategies in traditional factories are usually downloaded offline to programmable logic controllers (PLC), running on a closed control network, it is difficult to achieve flexible updates. The new type of PLC supports Artificial Intelligence (AI) modules, making it possible to use algorithms such as machine learning to achieve flexible self-organization of production tasks [2, 3].

The sample data of the machine learning algorithm comes from the control and state parameters generated by the field devices such as sensors, controllers and actuators in the historical production process. There are a large number of devices on the ground floor of the factory, and the data frame generated by a single device is very short, but due to the small generation cycle, a huge quantity of data is generated. These massive data are stored in cloud servers. If cloud-based data services are used, the data transmission delay will be very large [4]. However, industrial applications usually have strict requirements on the delay of data transmission. Therefore, the application of edge computing to the industrial IoT has great advantages [5]. The edge computing deploys server equipment at the edge close to the user, and uses its own storage and computing resources to provide low-latency data transmission services for user requests, and to provide guarantees for real-time task processing.

It has been discovered that online music and video, social networks, and other Internet material are the primary subjects of study approaches for caching strategies based on dynamic changes in popularity. More analytic characteristics are included in a particular network scenario to raise the popularity forecast accuracy and raise cache hits. However, the traffic and user request characteristics of industrial and Internet applications, such as social networks and online music and video, are entirely different. While the data volume created in the industrial production process is enormous, the data frame is short, and the timeliness is high, the audio and video data stream has the characteristics of a lengthy data frame, massive flow, and high bandwidth occupancy. Any user can request material from the Internet, and industrial edge cache nodes frequently cater to particular industrial control equipment. The production task is connected to the equipment’s content requirement. As such, edge caching in industrial application settings cannot be directly implemented using the current caching techniques.

However, the storage capacity of edge nodes is usually very limited, and it is impossible to cache all the content at edge nodes. Therefore, determining the content set to be cached through a reasonable caching strategy, maximizing the cache utilization, and reducing the number of content requests to the cloud server are very important for the service performance guarantee of the edge network.

Although there have been many research works on edge caching, as far as we know, there is no caching strategy for industrial edge computing applications.

The main contributions of this paper are in two aspects:

- An industrial edge network model and user request model are first established. A single-dimensional content popularity probability model is then proposed based on the feature changes of the content request sequence in the recent time window, and a new technique based on the content size cache replacement algorithm is then implemented. The industrial user requests are quantified based on shot noise model (SNM), and then establish a popularity change model of user requests;
- A new cache replacement algorithm is proposed, which comprehensively considers timeliness, content size priority and popularity prediction to determine the value of the content;
- The effectiveness of the proposed algorithm is verified by comparative experiments with five classical caching algorithms.

The remaining of the paper is organized as follows. In Section 2, the related work is discussed. In Section 3, the system model and problem formulation has been discussed. In Section 4, the user popularity prediction analysis is conducted. In Section 5, the proposed algorithm is presented. In Section 6, the performance evaluation is described. Section 7 discuss the limitations of the proposed method. Section 8 concludes the manuscript.

## 2 Related work

The success of an edge caching strategy mainly depends on its accuracy in estimating the value of cached content. The content popularity is an effective measure for making caching decisions. The classic Least Recently Used (LRU) algorithm [6] takes user access time as the popularity index, and the access content closest to the current moment has the highest popularity, which is easily disturbed by some accidental access content. The Least Frequently Used (LFU) algorithm [7] takes the access frequency of different contents as the popularity index, and the content with the most access times is the most popular, which can easily lead to the problem of cache pollution. The Size algorithm [8] takes the content size as the popularity index, and the smallest content has the highest popularity, which may cause frequent requests for large content with high popularity, resulting in a waste of bandwidth. In response to the problems of a single feature index, many studies have proposed a hybrid caching strategy based on multiple features such as access time, access frequency, and content size to improve the accuracy of popularity evaluation [9].

These traditional caching algorithms are easy to implement and have low algorithm complexity, and have been widely used. However, with the explosive growth of Internet data and the emergence of new networks such as edge computing, content-centric networks, and 5G networks, higher requirements have been placed on caching strategies. Therefore, research work in recent years has mainly proposed corresponding caching optimizations for new network scenarios. According to the relationship between the cached content set and the user requested content set, these caching strategies can be divided into two categories: 1) Assuming that network nodes cache all the content requested by users, this kind of caching research will mainly focus on how to cache the requested content on different nodes. The general approach is to transform the cache deployment problem into a linear optimization problem based on the network performance indicators such as cost and delay [10, 11], and then find an optimized global cache deployment scheme through evolutionary algorithms or heuristic algorithms. This kind of strategy needs to collect global network information, and the solution is expensive. Therefore, references [12, 13] uses the cooperative relationship between the nodes to propose a distributed cache strategy to obtain the best cache content set of nodes. 2) Caching research. It is assumed that the cache capacity is limited, and the network nodes only cache part of the content. The classic most-popular content (MPC) [14] algorithm believes that caching hot content is more efficient than caching non-hot content, so the popularity of each content is recorded through the popularity table value, and only that content is requested which has enough popularity.

Reference [15] comprehensively compared the performance of MPC, and proposed an improved algorithm for named data networks. Reference [16] proposed an improved MPC algorithm based on motion sensing for device-to-device (D2D) networks. Reference [17] proposed a distributed probabilistic caching strategy for the needs and preferences of vehicles in the Internet of Vehicles (IoV), as well as the relative positions of the sending and receiving nodes. Most of the current caching strategy research adopts a static content popularity model, which is usually assumed to obey the Zipf distribution [18–23]. However, the static model ignores the dynamism of the user’s request preference for different content changing with time in real scenarios. When the content request changes, the performance of the caching algorithm will degrade. Therefore, recent research has turned to analyzing and predicting the dynamics of popularity and designing dynamic caching strategies. These caching strategies mainly describe the popularity model from the characteristics of the content itself or the characteristics of user requests.

Some researchers analyze video and more relevant features of social content for popularity prediction. Some data show that video traffic data has become the main traffic of the Internet [24]. Reference [25] serialized video files into blocks with name prefixes and sequential indexes, and found the correlation features between the video blocks by analyzing the user’s request behavior to predict the popularity of future video blocks. Reference [26] analyzed the correlation between TV programs and features such as launch date, broadcast time, and program type, and extracted the key features that affect the popularity of programs to build a model of program popularity changing over time, using random forests. The algorithm constructs a TV program popularity prediction model, and proposes a program cache scheduling algorithm. Social traffic data is another type of active Internet traffic, which can be used to analyze the popularity of content by using the communication characteristics of social media. Reference [27] proposed an online popularity prediction method for microblog messages based on the propagation acceleration and user activity. Reference [28] proposed a pop caching strategy for social networks. The algorithm does not predict the individual popularity of each content, but assumes that the content popularity is similar when the context features are similar, and uses four historical visits as the current context feature.

Another group of researchers mines user preferences and request characteristics in new network scenarios for dynamic popularity prediction. For example, an important feature that distinguishes wireless networks from wired networks is the mobility of users, and different users have different spatial and temporal characteristics when they move. Therefore, many researchers have proposed a popularity prediction algorithm and a caching strategy for edge nodes for mobile edge computing applications. For example, references [29, 30] used the location features of edge nodes to represent user preferences at the location, and proposed an online prediction algorithm to improve the content hit rate. Reference [31] determined the user’s movement pattern through the base station to calculate the popularity of local content, and cached the files requested by users with different moving speeds in different base stations. Reference [32] established a Markov model for the mobile historical data of mobile network users to predict the behavior of users in terms of movement and requests, and pre-cached according to the calculated content popularity. For the time-varying and unknown content popularity, reference [33] used Bernoulli and Poisson as content request models, respectively, for small base station nodes in heterogeneous wireless networks, and proposed a method based on the estimated content popularity and optimal value. The error determines the cache update, and the simulation results show that the performance is better than the regular cache update.

A federated learning (FL) model for IoT anomaly detection was suggested in [34]. Only changed weights are shared in the central FL server, which aggregates the local models using a Mini batch average aggregation strategy to produce a global model. The data is stored on localized IoT devices for model training. To train the clients’ models with the new data instances, this global model with updated parameters is distributed among them. The important novelty is that this federated round is repeated for each client. The local model for anomaly prediction is based on deep neural network (DNN) prediction and mutual information-based feature selection. Reference [35] proposed the network training method to train the adaptive network and update the parameters, and the genetic algorithm (GA) and modified GA optimization approaches are used first. After that, a wind turbine pitch angle controller was created using this trained adaptive network. The findings of the suggested method indicate less error and training time in the intended results when compared to the results of typical parameter optimization methods. By using trained parameters as a control signal for pitch angle entering, the fluctuations of wind turbine mechanical power are also reduced. Reference [36] treated the task scheduling issue as an optimization problem taking into account higher QoS criteria for the implementation of IoT task requests in the fog-cloud environment. The task scheduling issue in the fog-cloud environment was resolved using a combination of the AO_AVOA and AVOA algorithms. The AO_AVOA was utilized to enhance the AVOA exploration process, and the AO algorithm’s exploration and AVOA’s exploitation phases were integrated. The scheduling problem was optimized using the optimizer technique to determine the optimum virtual machines for the fog-cloud environment by minimizing the makespan time function. Reference [37] provide a secure protocol to strengthen the RPL protocol’s defenses against various network threats. A key agreement protocol and authentication method based on the ECC theory are suggested for this purpose. Reference [38] presents a novel hybrid intelligence method called Grey Wolf Optimizer-Feature Weighted-Multiple Kernel-Support Vector Regression (GWO-FW-MKL-SVR). The most crucial TBM performance factors, including chamber earth pressure, total thrust, cutterhead torque, cutterhead speed, cohesion, internal friction angle, compression modulus, boulder ratio, uniaxial compressive strength, and rock quality designation, were measured and taken into account as model inputs for this project. Three models—biogeography-based optimization (BBO)-FW-MKL-SVR, MKL-SVR, and SVR—were also presented to forecast the TBM PR in order to demonstrate the GWO-FW-MKL-SVR model’s potential. Reference [39] suggest a hyperspectral evaluation approach for measuring salt-induced weathering on sandstone surfaces. Data collection for microscopic observations of sandstone in salt-induced weathering conditions and machine learning technologies for a predictive model make up our innovative approach.

It initially uses a near-infrared hyperspectral imaging approach to determine the microscopic morphology of sandstone surfaces. Then, based on investigations of spectral reflectance fluctuation, a salt-induced weathering reflectivity index is developed. The principle components analysis-Kmeans (PCA-Kmeans) algorithm is then used to fill in the gaps between the linked hyperspectral images and the salt-induced weathering degree.

In a study that comprehensively considers content file popularity prediction and caching system design, the authors in [40] studied how to predict each user’s preference for each content file through collaborative filtering and convert this matrix that reflects the popularity of content files is applied to the caching strategy of cellular networks, but the mobile characteristics of users make this method difficult to implement in actual systems. In addition, references [41, 42] uses online learning methods for active caching of content files. However, caching systems based on online algorithms will perform deployment updates of cached content files too frequently, resulting in excessive backhaul link overhead and additional load pressure on the system. In addition, due to the slow convergence speed of the online algorithm, it may not be able to cope with scenarios where the popularity of content files changes rapidly. On the other hand, deep learning (DL)-based methods bring new ideas and greater performance improvements to the performance optimization of mobile edge networks. Reference [43] applied the idea of deep reinforcement learning (DRL) to joint optimization of computing, caching and communication resources in mobile edge networks. Reference [44] use DNN to learn cached content distribution optimization algorithms to enable mobile edge networks to achieve cached content distribution with the lowest energy consumption.

Machine learning (ML) and Artificial Intelligence (AI) plays a vital role in industrial edge network optimization. Reference [45] proposed a content classifier was created, and the popularity of each content class was predicted using a streamlined bidirectional long short-term memory (Bi-LSTM) network. Reference [46] proposed a FL-based popularity prediction policy that takes advantage of user choice in context spaces that are adaptively partitioned. Reference [47] used Bayesian learning to model the content request pattern using a Poisson regressor that was based on a Gaussian process. To protect user data privacy, a proactive content caching system based on FL was proposed in [48]. To forecast the popularity of a piece of content, a probabilistic latent semantic analysis was used to model the user request behavior. In [49], the authors looked at the issue of predicting the popularity of contents in fog radio access networks (F-RANs) and employed DL for processing. It has been demonstrated that using reinforcement learning, the best cache replacement strategy may be discovered in order to maximize the cache hit ratio.

Data security and privacy is one of the most important aspects of industrial IoT. Reference [50] reviews the typical system SCADA in industrial control environments, and summarizes 24 risk assessment methods in industrial control environments, based on their respective assessment goals, application scenarios, and risks. Management and risk objects demonstrated the necessity of the above risk assessment and proposed several research hotspots, especially emphasizing the risk assessment of control flow hijacking caused by information leakage in SCADA systems. From the perspective of intrusion detection, reference [51] achieved Intrusion modeling and real-time detection of industrial control environments represented by PLC can locate important intrusion behavior characteristics by extracting key information in transmission private protocols. The same intrusion detection method is implemented in [52], which is targeted at medical industrial control system. It proposes the characteristic definition of behavioral rules and a behavioral evolution model in the context of a state machine, thereby equating the attacker’s malicious behavior detection to the illegal transfer measurement of target information in the behavioral rules and state machine in this environment. Reference [53] analyzes the industrial control system from the perspective of attack effect assessment, take the common ModBus protocol as an example, design a threshold measurement method, and successfully detect hidden attack. As for the underlying device firmware in the industrial control environment, there is a lot of work around control conduct research on the measurement and mining of key information and data flow. The authors in [54] proposes C-FLAT that performs remote security proof of control flow for embedded systems at the device layer, and designs controls for basic control flow runtime blocks in a black box flow security verification method, and finally design a prototype system based on ARM’s trusted base. Reference [55] is to carry out backdoor and vulnerability mining for the web layer services provided by the firmware image, extract the web-related file structure in the firmware through automated firmware unpacking methods, and analyze several key ones based on customized Web vulnerability type characteristics. The sampling points of the data are finally given to determine the vulnerability mode, and the vulnerability exploit program is automatically generated. A large number of unknown backdoors and vulnerabilities are obtained in its experimental analysis, but the applicability of its automated firmware unpacking analysis method is not strong at present, and key data extraction is limited to web types. Reference [56] mainly deploys function-level similarity recognition for binary data in device layer firmware, and successfully achieves compatibility with x86, ARM, and MIPS while shielding the CPU architecture. This method has verified known backdoors on a large number of platforms. The Avatar [57] tool directly conducts offline analysis of control flow and data flow for firmware devices, and completes a combined static and dynamic analysis method based on dynamic symbolic execution and QEMU virtual machine execution, which can realize the mining of vulnerabilities and backdoors, but the execution efficiency is low. Reference [58] also focuses on the ciphertext search scheme classified research, and expanded the description of privacy protection technology based on access control and hidden access mode, and finally proposed a privacy protection research direction for search data protection. References [59, 60] proposed security analysis and practicality for the application scenarios of the industrial IoT. The security data protection method provides a security threat assessment method in actual application scenarios, defines the target data set, and proposes a low-overhead protection framework. In addition, from the perspective of location privacy data protection of the IoT, reference [61] proposed location service application scenarios, system framework, etc., and then the attacker model and privacy protection metrics in LBS are discussed, and finally potential research directions for future LBS privacy protection technology are given. Reference [62] relies on mobile devices sensor on the mobile phone explores the derivation method of the user’s location and path, and aggregates the data in the sensor to design a semantic aggregation algorithm of the user’s location and user characteristics rules, which poses a challenge to the location privacy protection on the mobile device from the perspective of attack. The two-layer transmission privacy protection in the sensor network environment is also one of the research hotspots. References [63, 64] respectively provide privacy protection methods for two-layer sensor networks, and design query processing algorithms based on *k*-NN and Top-*k* methods respectively. The former is aimed at Node capture attacks and collusion attacks achieve better defense effects, and the latter provides a security proof process and energy consumption analysis process. Reference [65] examines how to reduce bandwidth waste in relation to the distributed deployment of application components. However, from the perspective of power consumption control, if all of an application’s components are put on a single node, the service reliability suffers. As a result, a technique is offered to address single points of failure and boost application dependability against failure. Reference [66] considers the fault tolerance threshold as a criterion to ensure the dependability of applications in operation. It presents the deployment of modules for IoT applications on fog infrastructure as a multi-objective optimization problem that minimizes power usage and bandwidth waste. A multi-objective optimization genetic algorithm (MOGA) is developed to address this combinatorial issue. It takes into account the application’s QoS and dependability in its constraints, as well as the physical resource usage and bandwidth waste rate in its objective functions.

It can be found that the research methods of caching strategies based on dynamic changes of popularity are mainly for online audio and video, social networks and other Internet content. In a specific network situation, more analysis features are introduced to improve the accuracy of popularity prediction, thereby improving cache hits. However, industrial and Internet applications such as online audio and video and social networks have completely different traffic and user request characteristics. The audio and video data stream has the characteristics of long data frame, large flow and high bandwidth occupation, while the data volume generated in the industrial production process is huge, the data frame is short and the timeliness is high. The content in the Internet may be requested by any user, and the industrial edge cache nodes often serve specific industrial control equipment. The content request of the equipment is related to the production task. Therefore, the existing caching algorithms cannot be directly applied to edge caching in industrial application scenarios.

Table 1 compares the performance of the proposed algorithm with state of art algorithms. As can be seen from Table 1, the performance of the proposed algorithm is better than existing algorithm which validated its effectiveness.

**Table 1:** Comparison of proposed and state of the art algorithms.

Reference	Objective	Approach	Infrastructure	Proactive	Cooperative	Domain
[32]	Delay, hit rate	Markov process	x	x	x	✓
[35]	Response time	Genetic algorithm		x	x	x
[37]	Hit rate	Elliptic-curve cryptography	x	✓	x	x
[39]	Delay	Machine learning	x	x	✓	✓
[41]	Transmission overhead	Online learning algorithm	x	x	✓	✓
[44]	Backhaul load	Deep neural network	x	✓	x	x
[47]	Delay	Bayesian learning	✓	x	x	✓
[49]	Hit rate	Deep learning	x	x	✓	x
[51]	Traffic load	Neural network	x	x	✓	✓
[53]	Delay	Regression	x	✓	x	x
[56]	Delay	Machine learning	x	x	x	✓
[58]	Hit rate	Hidden access mode	x	✓	✓	x
[60]	Delay	Machine learning	x	x	x	✓
[64]	Hit rate, delay	Bayesian learning	x	x	✓	✓
Proposed	Cache hit rate, transmission delay, prioritize control data	Content popularity probability model	✓	✓	✓	✓

## 3 System model

This section builds network model and cache problem model for typical factory application requirements.

### 3.1 Network model

**Fig 1:**
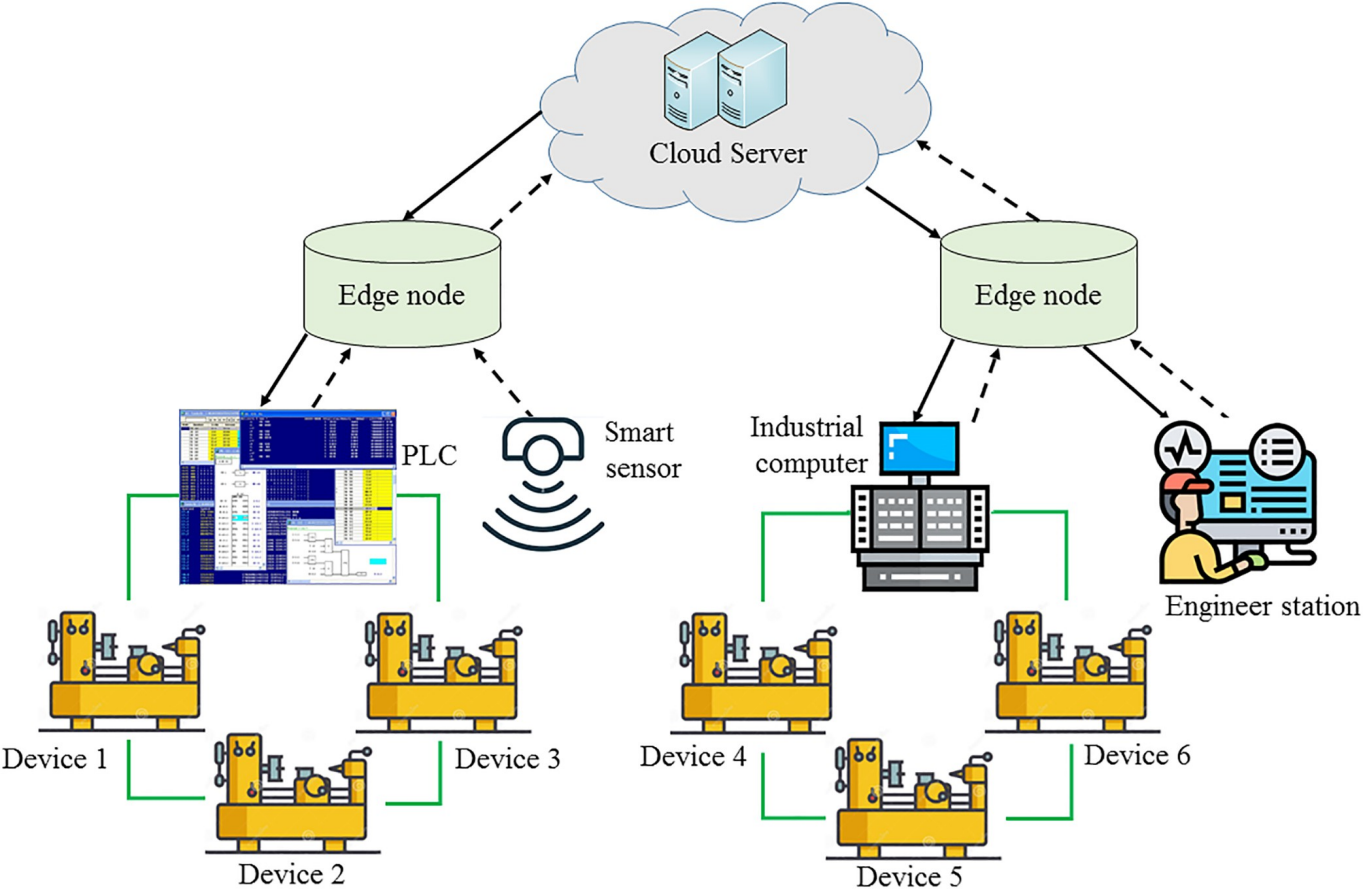
Proposed industrial network architecture.

The edge network architecture in a typical industrial application scenario is shown in Fig 1. The top layer is the cloud server center. The middle layer is the edge node layer, and each edge node has a limited size of cache resources [65, 66]. The bottom layer is the field intelligent equipment, such as intelligent sensor, PLC, industrial computer, engineer station, etc.

Assume that the set of historical production control and state parameters generated by field devices such as the underlying sensors, controllers and actuators is denoted as Ω. When the field device performs the learning task [67–69], it will request a part of the content set *M*, the size is |*M*|, and the size of each piece of content *m* is *S*_*m*_. In order to reduce the data transmission delay, *N* edge nodes will provide cache service, and the cache capacity of each edge node *n* is *L* (unit is MB).

### 3.2 Problem model

Assuming that the industrial user has issued *K* content requests in total, *C*_*mk*_ indicates whether the content requested for the *k*th time is the *m*th content, if yes, then *C*_*mk*_ = 1, otherwise *C*_*mk*_ = 0. *A*_*nk*_ indicates whether the requested content is cached to the edge node *n*, if so, *A*_*nk*_ = 1, otherwise *A*_*nk*_ = 0. The cache hit ratio is expressed as

$$H=\frac{1}{K}\sum_{n=1}^{N}\sum_{k=1}^{K}A_{nk}$$ (1)

The data transmission delay in the edge network consists of two parts. If the user requests the content to be cached, the delay is the time for the content to be transmitted from the edge node to the user, that is, the delay is the cache read time *d*. If the content requested by the user is not in the cache, the edge cache device needs to request the factory private cloud to cache the content [28, 70, 71], and the data transmission will be limited by the capacity of the backhaul link. *γ* represents the transmission factor of the link, that is, the delay caused by network instability or congestion. Then, the average transmission delay *D* can be expressed as:

$$D=\frac{1}{K}\sum_{n=1}^{N}\sum_{k=1}^{K}\left[\left(1-A_{nk}\right)\times \frac{1}{B}\left(\gamma +\sum_{n=1}^{N}\sum_{k=1}^{K}C_{mk}\times S_{m}\right)+A_{nk}\times d\right]$$ (2)

Where, *A*_*nk*_ represent the condition that the requested content is cached to the edge node *n*, if so, *A*_*nk*_ = 1, otherwise *A*_*nk*_ = 0. *B* is the bandwidth from transmission chain to the edge node. *γ* represents the transmission factor of the link, that is, the delay caused by network instability or congestion [72–74]. *S*_*m*_ denotes the leased cache capacity, whereas *C*_*mk*_ is explained in Eq (1).

The optimization goal of this paper is to maximize the cache hit rate of edge nodes, minimize the transmission delay of user requested content, and prioritize control data. The optimization problem can be formulated as:

maxH (3)

minD (4)

The above constraints are based on multi-objective optimization problem. In multi-objective optimization, there are more than one objective so that objectives may have conflicting [65, 75, 76]. Thus, sub-optimal solutions are obtained. To evaluate which sub-optimal solution has superiority over its set of sub-optimal solutions, the concept of ‘dominance’ is defined. Let *H* = [*h*_1_, *h*_2_, …, *h*_*M*_], and *D* = [*d*_1_, *d*_2_, …, *h*_*M*_] be two M-dimensional vectors [77]. The dominance is expressed as

HdominatesDorDisdominatedbyH:H≤D (4a)

$$\text{if}\;\text{and}\;\text{only}\;\text{if}:\forall \;k\in \left\{1,2,\ldots ,M\right\},\;h_{k}\leq d_{k}\;\;\text{and}\;\text{for}\;\text{at}\;\text{least}\;\text{one}\;k\in \left\{1,2,\ldots ,M\right\},\;h_{k}<d_{k}$$ (4b)

There exist non-dominated solutions (Pareto optimal solutions) which is determined from using the method [68, 78, 79].

## 4 User request popularity prediction

This section first combines the characteristics of user requests in industrial applications to establish a user request model, then sorts out the feature attributes of industrial user request content [80–82], and gives a prediction method for the change of user request popularity.

### 4.1 User request model

At present, the most widely used user request model is the Independent Reference Model (IRM) [65]. As a static model, the IRM model assumes that the popularity of content requests does not change with time, and user requests follow the Zipf distribution [83]. This model simplifies the complexity of the caching problem, but cannot reflect the temporal locality of content popularity. In industrial applications, the content requested by the device changes with the change of production tasks, and the life cycle of the requested content is closely related to the production task, so the IRM model is not suitable for industrial scenarios. This paper adopts the SNM proposed by [66] as the user request model. Compared with the IRM model, the SNM model describes the content request process and can better represent the dynamic trend of different content popularity over time.

Under the SNM model, the content request generation process is assumed to be a Poisson process, and the request process for each content is an independent homogeneous Poisson process. The entire request process is represented as the superposition of many independent processes, and each content request process corresponds to an independent process [84, 85]. Specifically, for content *m*, the request arrival rate at time *u* is expressed as:

$$Y_{m}\left(u\right)=V_{m}\lambda_{m}\left(u-\tau_{m}\right)$$ (5)

Among them, *m* represents the time point at which content *m* enters the system, and *λ*_*m*_ (*u*) represents the law that the arrival rate of requests for content *m* changes with time *u*, that is, the “popularity profile”. In [67], three popular profiles are mentioned as exponential profile [86–88], uniform profile and random profile. To simplify the application, this paper adopts a uniform popularity profile, that is, the content *m* follows a homogeneous Poisson process with an average arrival rate *λ* in the life cycle *T*. The average amount of content requested during the entire evaluation time *E* is *λE*. *V*_*m*_ represents the total number of times the content *m* is requested in the active time. The above features are used to describe the request process of the content *m* changing with time [68, 89, 90].

**Fig 2:**
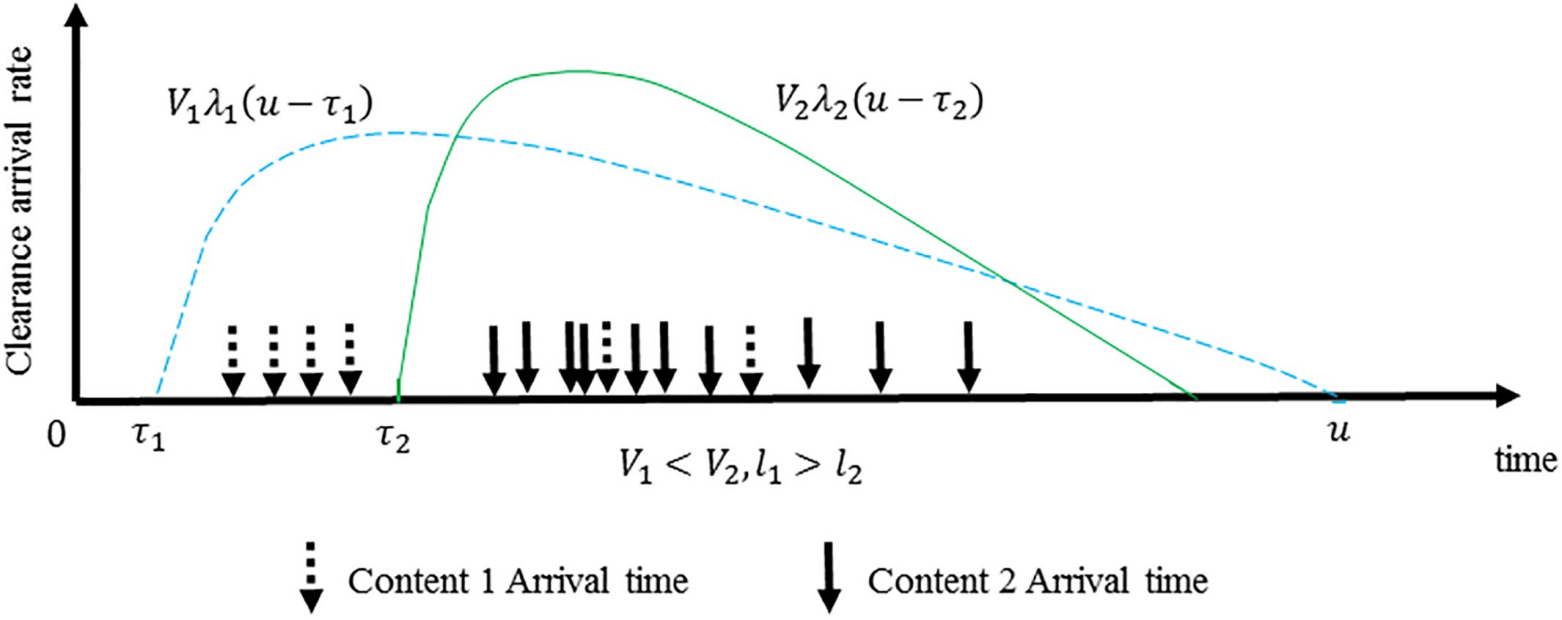
Illustrations of different content requests.

Fig 2 shows an example of two different contents request processes. *V*_1_ represents the average number of requests for content 1, *u* represents the current time, *τ*_1_ represents the moment when content 1 first arrived, *l*_1_ represents the generation period of content 1, *λ*_1_ (*u*) is a value that changes with time which satisfy $\int_{0}^{\infty }\lambda_{1}(u)du$, so *V*_1_ *λ*_1_ (*u*-*τ*_1_) represents the instantaneous rate of arrival of content 1 requests, each arc is a Poisson process for a certain content [91–93], and multiple content-independent Poisson processes are superimposed. Thus, the SNM model is realized.

### 4.2 Prediction of changes in popularity of user requests

The dynamics of equipment requests in industrial applications is highly correlated with the current production task update adjustment. An industry may have several different production optimization tasks happening in a day [69]. Taking the self-organizing scheduling of production as an example, the production line may contain multiple different types of equipment, and the content requested by the controller has the same location attribute even though it comes from different equipment in the same production line. Another example is that the controller optimizes the equipment fault prediction strategy based on the ML algorithm, and needs to request the relevant sensor historical data, historical diagnosis data and historical data of similar equipment that come with the equipment object [94, 95]. The content of these requests comes from the same type of object device. Through the comparison of these two practical application cases, it can be found that: When different tasks occur, the controller needs to request completely different content to run the learning algorithm. However, the content requested by each device has certain common characteristics, and these characteristics reflect the different production tasks currently in progress.

To summarize its characteristics, we define multi-dimensional feature attributes of content from industrial sites: time attribute, location attribute, source equipment attribute, object equipment attribute, etc. The time attribute is the occurrence time of the data collection. The location attribute is the place where data collection occurs; the source device attribute is the data source [96–98]. The object device attribute is the monitoring object of the data. For any content *m*, there are *W* characteristic attributes, which are represented by the set {*f*_*m*1_, *f*_*m*2_, …, *f*_*mW*_}.

Since the content is only active in a limited life cycle, the popularity analysis only counts the characteristic attribute changes of the requested content in the most recent time window. Suppose *T* is the size of the periodic time window, and *F*(*t*) is the feature set of all requested contents in the *t*-th time window. Using the Jaccard similarity coefficient to define the similarity function of the set of requested content in the *t*-th and (*t*−1)th time windows as [99, 100]:

$$Sim\left(t\right)=\frac{\left|F\left(t\right)\cap F\left(t-1\right)\right|}{\left|F\left(t\right)\cup F\left(t-1\right)\right|}$$ (6)

In the *t*-th time window, when the value of the similarity coefficient *Sim*(*t*) is greater than the threshold *θ*, it is considered that the production optimization task performed in the two time windows before and after has not changed. When the similarity coefficient value is less than the threshold *θ*, it is considered that the currently active production optimization task has changed [70, 101].

During the experiment, it is found that when the production optimization task changes, there is often a dominant content attribute characteristic that changes accordingly. Therefore, this paper does not count all the feature attributes, but uses the most frequent hotspot feature attributes in the recent period window to represent the current real-time task. The advantage of this single feature attribute indication is that it simplifies the calculation and reduces the complexity of the caching algorithm. Defining *M*_*o*_ (*F*(*t*)) as a hot spot feature attribute, the element ordinal number of *M*_*o*_ (*F*(*t*)) in the *F*(*t*) set is w* [88–90], that is, *f*_w*_ (*t*) = *M*_*o*_ (*F*(*t*)) (t), then similar calculation of the degree function can be simplified as:

$$Sim\left(t\right)=\frac{\left|f_{w^{*}}\left(t\right)\cap f_{w^{*}}\left(t-1\right)\right|}{\left|f_{w^{*}}\left(t\right)\cup f_{w^{*}}\left(t-1\right)\right|}$$ (7)

## 5 Proposed algorithm

Based on the prediction method of popularity change of user requests in Section 4.2, a caching algorithm based on periodic popularity prediction and size caching (PPPS) is proposed. On the one hand, the proposed algorithm considers the influence of popularity and content size, and sets the cached content value for each content [102–104]. On the other hand, the feature attribute content with high heat is stored in the free space of the edge cache in advance, which improves the hit rate of the edge cache and improves the cache utilization efficiency.

The proposed algorithm defines the cache value *Q* for each content, and uses the cache value function as the basis for cache replacement. The cache value function first considers the impact of time locality and sets the cache update time. Secondly, according to the recent historical request content, the popularity probability of each cached content is predicted. Furthermore, in industrial applications, the content of control and status information is small in size and high in frequency, and the content of audio and video is large but has low frequency. For any content *m*, get its value function *Q*_*m*_:

$$Q_{m}=\frac{t+p_{m}\left(t\right)}{S_{m}}$$ (8)

The size of the function value is negatively related to the probability that the cache content is replaced. Among them, *p*_*m*_ (*t*) represents the popularity probability of a single content *m* in the *t*-th time window. The hotspot attribute feature set of the content requested by the user in the *t*-th time window is {*f*_xw*_, *f*_(*x* + 1)w*_, …, *f*_(x + T)w*_}, and the popularity probability of the content *m* can be characterized as its feature attribute in the tth time window. The probability of occurrence of *t* time windows is:

$$p_{m}\left(t\right)=\frac{1}{\left|F\left(t\right)\right|}\sum_{x=1}^{T}\left(f_{xw^{*}}==f_{mw^{*}}\right)$$ (9)

The algorithm implementation process is as follows: Every time when a new user requests content arrives, it is judged whether the currently requested content already exists in the cache. If hit, update the value of the content. If it is not hit, it is judged whether the remaining space in the cache is enough to store the content. If yes, then it is directly stored in the cache queue. Otherwise, the content with the smallest cache value is replaced until the space is large enough to accommodate the new content [105]. Then judge whether the cumulative number of user requests reaches *T*, if so, update the value of *t*, *t* = *t* + 1. Simultaneously calculate the similarity coefficient *Sim*(*t*) of the requested content in the time window of *t* and *t* − 1. When it is less than the threshold *θ*, delete the non-hot feature attribute content in the cache queue, and randomly select the content of the hot feature attribute and put it into the cache. But the cache content value *Q* = 0. The pseudo code of the proposed PPPS algorithm implementation is shown in Algorithm 1.

**Algorithm 1:** Periodic popularity prediction and cache size

**Input:** User requests content set *K*

**Output:** Cache hit ratio *H*

  1: Initialize *j* = 0, *t* = 0

  2: For *k* = 1, 2, … *K* do

  3: User requests content *m*

  4: if *m* has been cached

  5: *j* = *j* + 1

  6: $\;Q_{m}=\frac{t+p_{m}}{S_{m}}$

  7: Else

  8: while (cache free size < *S*_*m*_)

  9: Delete the content of the minimum value *Q*_min_

  10: end while

  11: Cache file *m*, $Q_{m}=\frac{t+p_{m}}{S_{m}}$

  12: end if

  13: if mod (*k*, *T*) = = 0

  14: *t* = *t* + 1

  15: $Sim\left(t\right)=\frac{\left|f_{w^{*}}\left(t\right)\cap f_{w^{*}}\left(t-1\right)\right|}{\left|f_{w^{*}}\left(t\right)\cup f_{w^{*}}\left(t-1\right)\right|}$

  16: if *Sim* < *θ*

  17: Delete the content of *f*_w*_ ≠ *M*_*o*_ (*F*(*T*)) in the cache queue

  18: Randomly cache a batch of content whose feature attribute is *M*_*o*_ (*F*(*T*)), and its value *Q* = 0

  19: end if

  20: end if

  21: end for

  22: return $H=\frac{j}{K}$

### 5.1 Complexity analysis

The calculation of the time complexity of the proposed algorithm can be divided into two parts: cache update and content popularity calculation. The cache update process is similar to the LRU algorithm, and the algorithm complexity is O(1). The calculation of popularity mainly depends on the update of the hotspot feature attribute w*, which can be reduced to the problem of solving the mode of the *F*(*T*) set, so the algorithm complexity is O(TlogT) [106–108]. Combining these two parts, the time complexity of proposed algorithm is O(TlogT).

## 6 Performance evaluation

This section first introduces the experimental parameter settings, and then analyzes and compares the performance of the algorithm through three groups of experimental scenarios.

### 6.1 Experimental parameter settings

The simulations analysis is conducted using MATLAB R2017b, the operating system is Windows 10, the computer processor is Inteli7-8565U8 core, the main frequency is 1.80GHz, and the memory is 8GB. The simulation parameters are listed in Table 2.

**Table 2:** Simulation parameters.

Parameter	Value
Capacity of each cache device	20~180MB
Link transmission bandwidth from the cloud server to the edge node	100MBps
Delay factor of the buffered transmission link	15ms
Cloud server content storage capacity *M*	100 ~ 500
Threshold *θ*	0.1
Time window period *T*	100 ms
*α*	0.7
Total number of requests	19562
Content distribution model	Zipf distribution

The industrial edge network adopts the network structure of Fig 1. The top cloud stores everything. The edge nodes are deployed at the edge of the network, and each node has a limited size of cache space [109, 110].

Since there is no real data in an industrial environment, we generate content and user request sequences through simulation. The generated content size is a random number that obeys the Zipf distribution. It randomly generates content request data based on the SNM model. The life cycle of each content and the active start time of each content are randomly generated, and the location attributes of the content are divided according to the start time [111–113]. It is worth noting that the time window period *T* setting is related to the update frequency of production tasks. If the value of *T* is too small, the cache value will be updated frequently, which will affect the performance of the algorithm. There are many factors affecting cache performance, such as user request sequence, content size distribution, content type, cache capacity, etc. Three experiments were conducted to analyze the influence of the main factors on the performance of the algorithm [114].

#### Experiment 1

The performance of each algorithm with the cache capacity is compared under the data traffic generated by the IRM model and the SNM model. The purpose of this experiment is to explore the performance of the caching algorithm under the data traffic generated by different user request models. The experimental parameters are set as: The content size setting obeys the Zipf distribution, the parameter *α* = 0.7, the range is 1~5MB, the content type *M* = 200, the total number of requests is 19562 times, and the cache space is 20~180MB.

#### Experiment 2

The performance of the caching algorithm when the content size is the same is studied, and the effect of the content size on the caching algorithm is explored in comparative Experiment 1. The content size of Experiment 1 is to consider the industrial network mixed with various types of content such as control data, video data, and image data [115]. The content size setting of Experiment 2 actually has a realistic application background. In many traditional factories, the data type in the industrial network is single, and the field device generates a short control data frame, which is uploaded to the cloud server through the gateway cache, showing a single content size. The experimental parameters are set as follows: the size of all content is set to 1 MB, the content type *M* = 200, the total number of requests is 19562 times, and the cache space is 20–180 MB.

#### Experiment 3

The cache capacity is fixed, and the effect of content type on the performance of the cache algorithm is mainly studied. The experimental parameters are set as: The fixed cache is 300 MB, the content type *M* is 100~500, and each content type is the corresponding number of requests is 8810, 19562, 29076, 38875, 49356, the content size is randomly generated, obeys the Zipf distribution, the parameter *α* = 0.7, and the range is 1~5 MB [116].

To better analyze and compare the algorithms, this paper implements five classical caching strategies FIFO, LRU, LFU, GDS, and MPC algorithms as comparison algorithms. Among them, the MPC algorithm itself does not consider the limited cache capacity, so many research works combine the MPC algorithm with the LRU to realize the replacement of the cache content. This paper adopts the same strategy, and the popularity threshold of the MPC algorithm is set to 3 [117]. The cache hit rate and average delay are used as the performance evaluation indicators of the algorithm.

### 6.2 Simulation results

**Fig 3:**
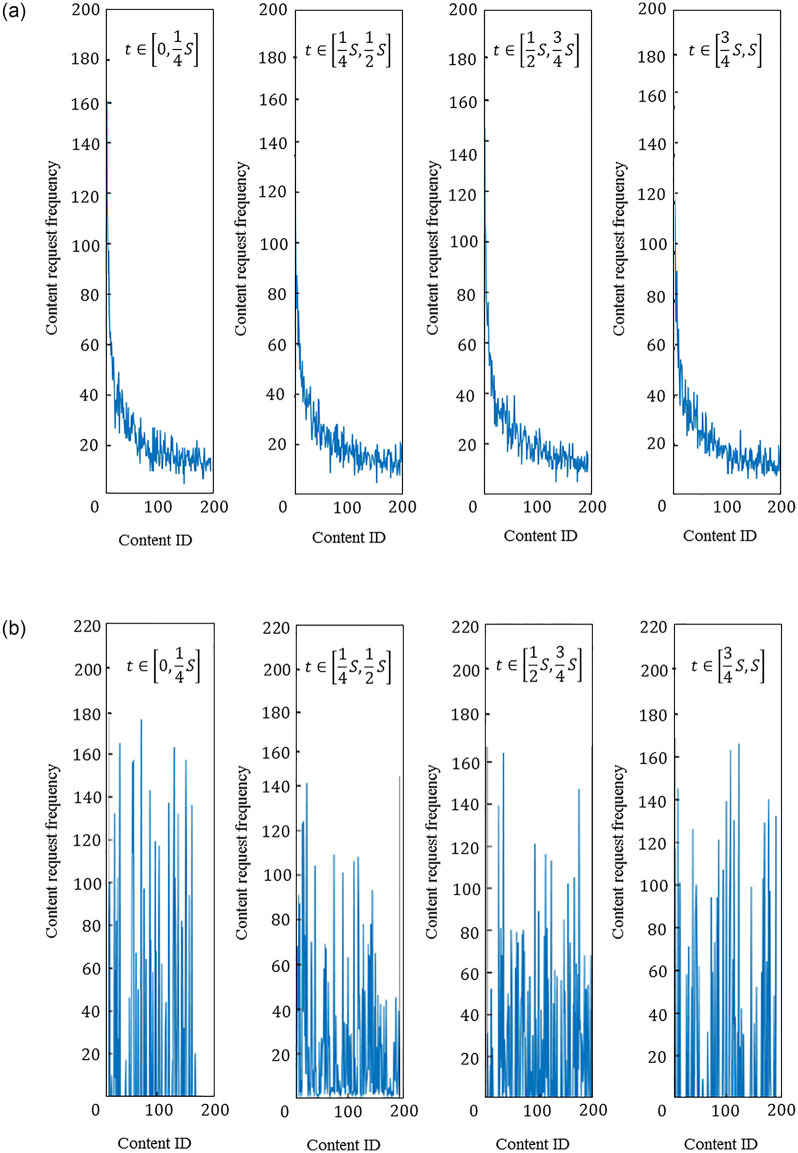
Comparison of content request of both models. (a) IRM, (b) SNM.

1) User request impact analysis of IRM model and SNM model. The SNM model and the IRM model are used to generate user request sequences respectively. Assuming that the total request time is *S*, it is divided into four equal parts in chronological order. Fig 3 compares the content requests under the two models. The frequency of requests at different time periods in the process. The content sequence generated by the IRM model follows the Zipf distribution, and the content popularity remains unchanged. The content sequence generated by the SNM model can better reflect the dynamic changes of content popularity in the life cycle [118]. With the gradual increase in the popularity of different content, the hot content has a peak request in a certain period of time, which is in line with industrial application scenarios.

**Fig 4:**
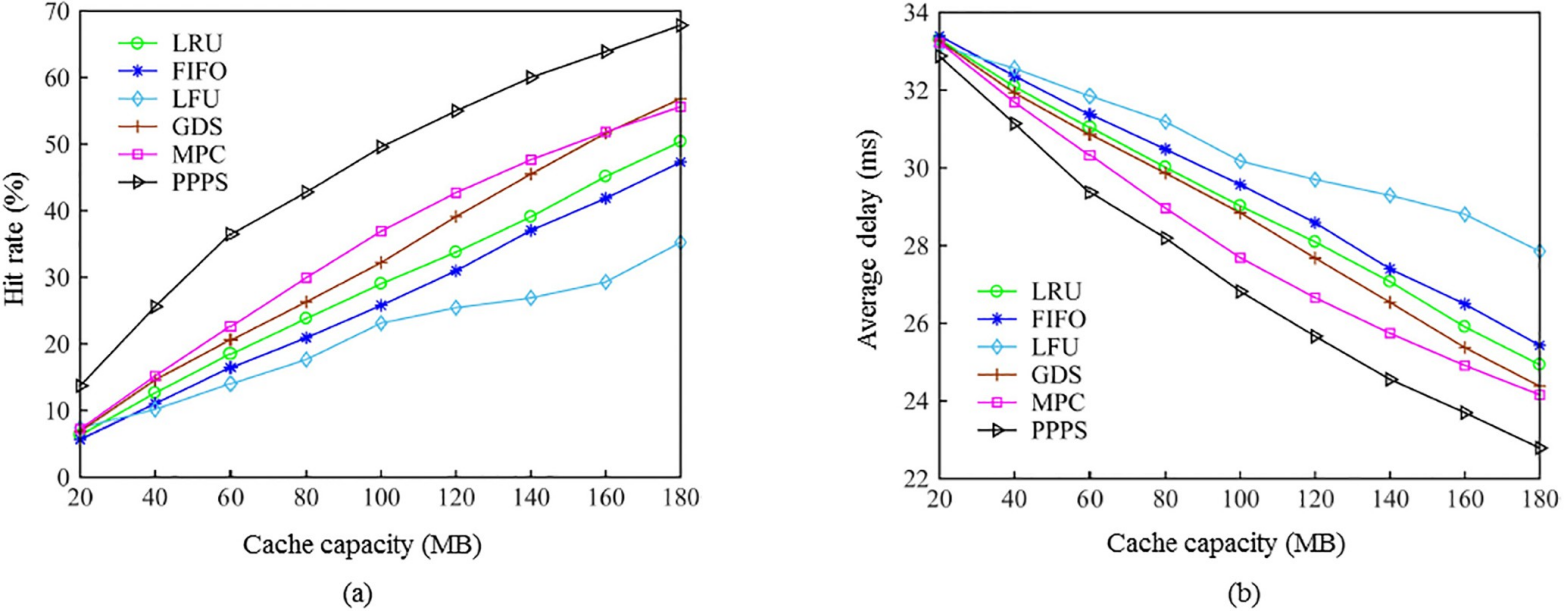
Comparison of the hit rate and average delay of the proposed and existing algorithms using the SNM model. (a) variation of hit rate with cache capacity. (b) variation of average delay with cache capacity.

**Fig 5:**
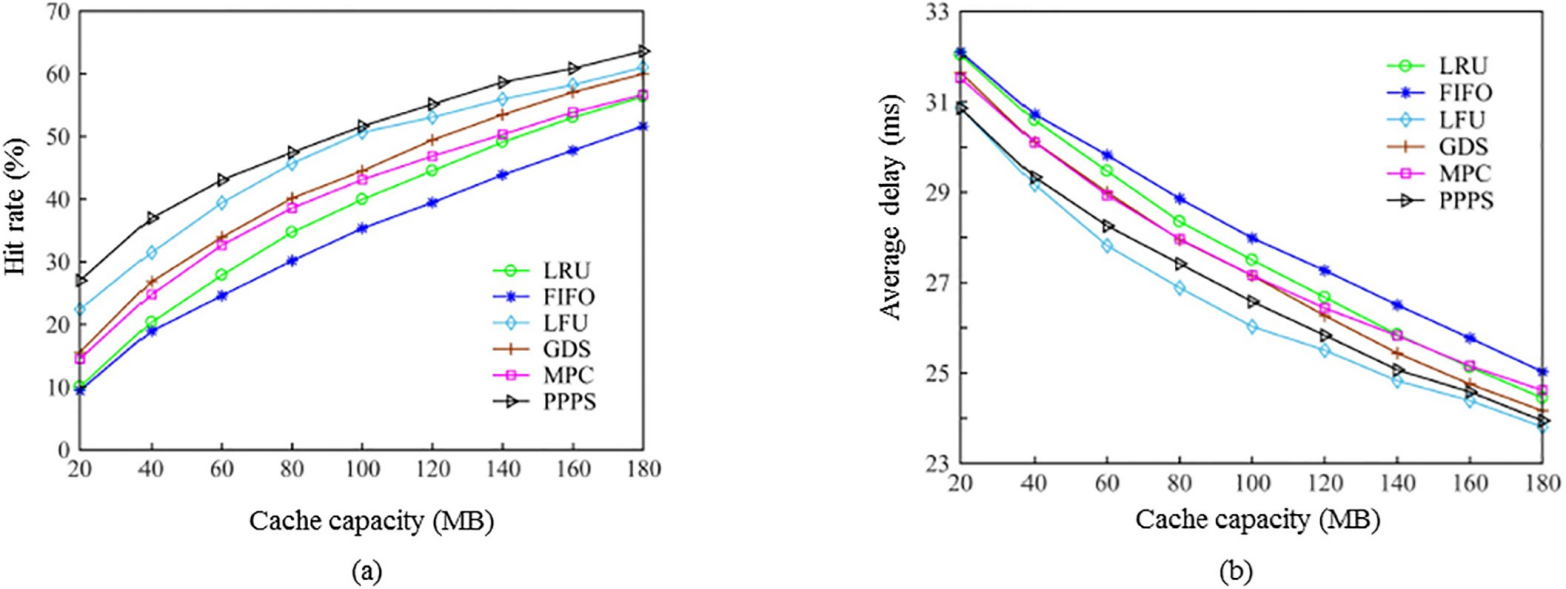
Comparison of the hit rate and average delay of the proposed and existing algorithms using the IRM model. (a) variation of hit rate with cache capacity. (b) variation of average delay with cache capacity.

Figs 4 and 5 analyze the performance comparison of Experiment 1 for algorithms under SNM and IRM models. First, Fig 4 describes the performance of the proposed algorithm and the five contrasting algorithms under the user request sequence based on the SNM model, the hit rate and the average delay as the cache capacity changes. It can be seen from Fig 4(a) that the LFU algorithm has the worst hit rate, which is caused by the “cache pollution” of the content that has been outdated for a long time in the cache. The hit rate of the FIFO and the LRU algorithms is second, reflecting that the two algorithms have poor adaptability to the content request distribution under the dynamic model. The MPC algorithm only caches popular content that exceeds the threshold, so when the cache capacity is small, the performance of MPC on the basis of LRU has a certain improvement, but as the cache capacity increases, the hit rate tends to converge. The content size and access time are weighed in the graph data science (GDS) algorithm, so it is more suitable for industrial applications where there is many small-sized control traffic. The performance of GDS algorithm is close to that of MPC when the cache capacity is small, but with the increase of cache capacity, the GDS can achieve a better hit rate than MPC. The proposed algorithm has the highest hit rate all the time. When the cache capacity is 120 MB, the hit rate of the proposed algorithm is 12.3% higher than MPC, 15.7% higher than GDS, 21.3% higher than LRU, 24% higher than LFU, and 29.6% higher than FIFO.

Fig 4(b) depicts the average delay variation of the algorithms with the change of cache capacity. It can be seen that the change of the average delay is opposite to the change of the hit rate. With the increase of the cache capacity, the delay of the algorithms gradually decreases and the rate performance is consistent. When the cache capacity is 120 MB, the average delay of the proposed algorithm is 12.3% lower than MPC, 15.7% lower than GDS, 21.3% lower than LRU, 24% lower than LFU, and 29.6% lower than FIFO. Therefore, it can be said that the proposed algorithm balances the influencing factors such as content size, time and popularity, and has the best performance when the data request is dynamically distributed.

Fig 5 shows the comparison of the hit rate and average delay of the algorithms with the change of cache capacity when the content request sequence follows the Zipf distribution. It can be seen that the LFU algorithm has a high hit rate, because the Zipf distribution follows the law of 28, and a small amount of content is requested multiple times. This is for the LFU algorithm that uses the request frequency as the popularity indicator very beneficial. As the cache capacity increases, the hit rate of the proposed algorithm is always higher than that of the other five algorithms. When the cache capacity is 120 MB, the hit rate of the proposed algorithm is 9.8% higher than that of MPC, 5.6% higher than that of GDS, 2.2% higher than that of LFU, 11.5% higher than that of LRU, and 15.6% higher than that of FIFO. On the average delay performance, the proposed algorithm also has lower latency [119]. Therefore, the proposed algorithm also has relatively stable performance when the data request is Zipf distribution.

**Fig 6:**
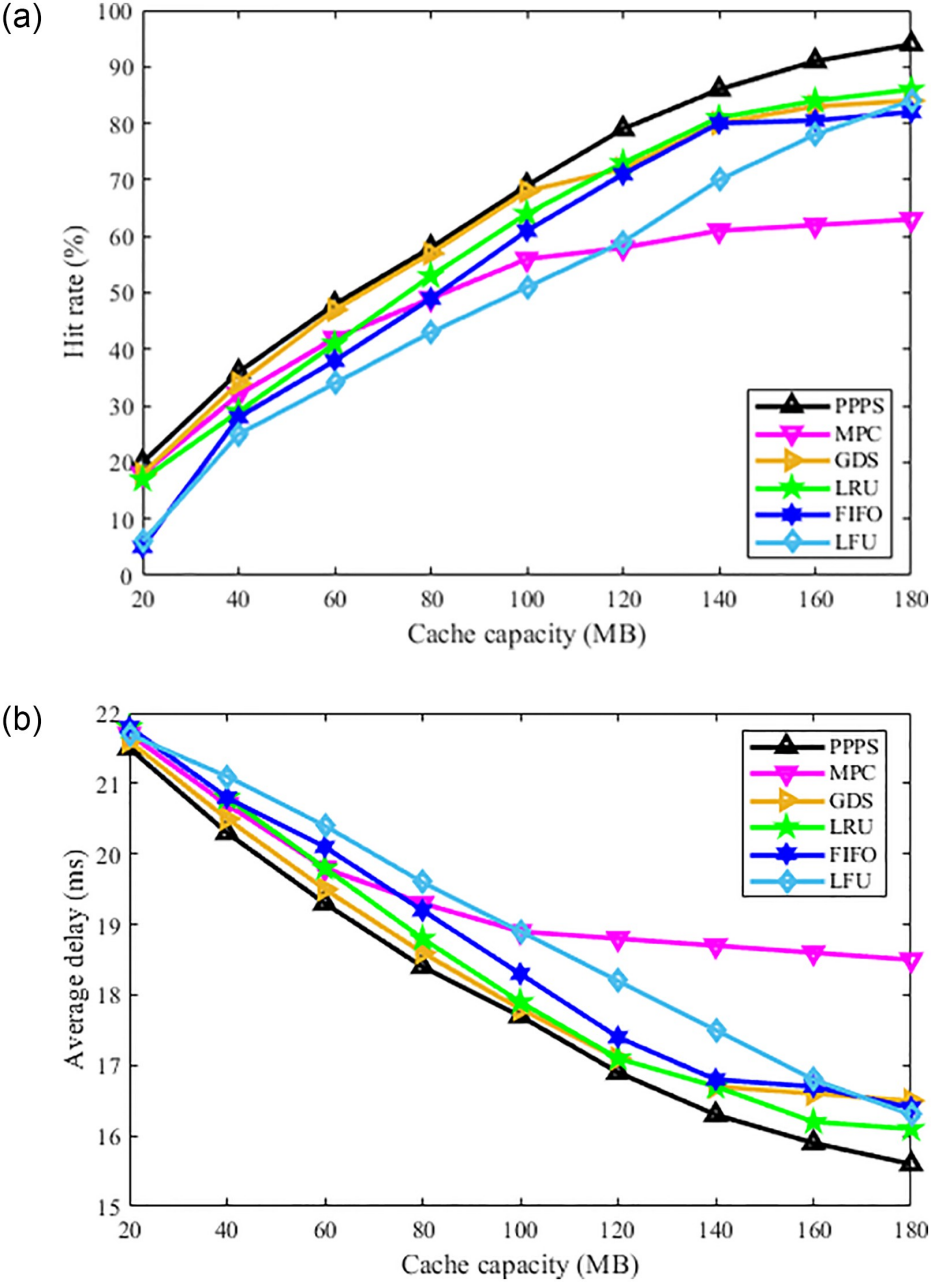
Comparison of the hit rate and average delay of the algorithms with same size content. (a) variation of hit rate with cache capacity, (b) variation of average delay with cache capacity.

**Fig 7:**
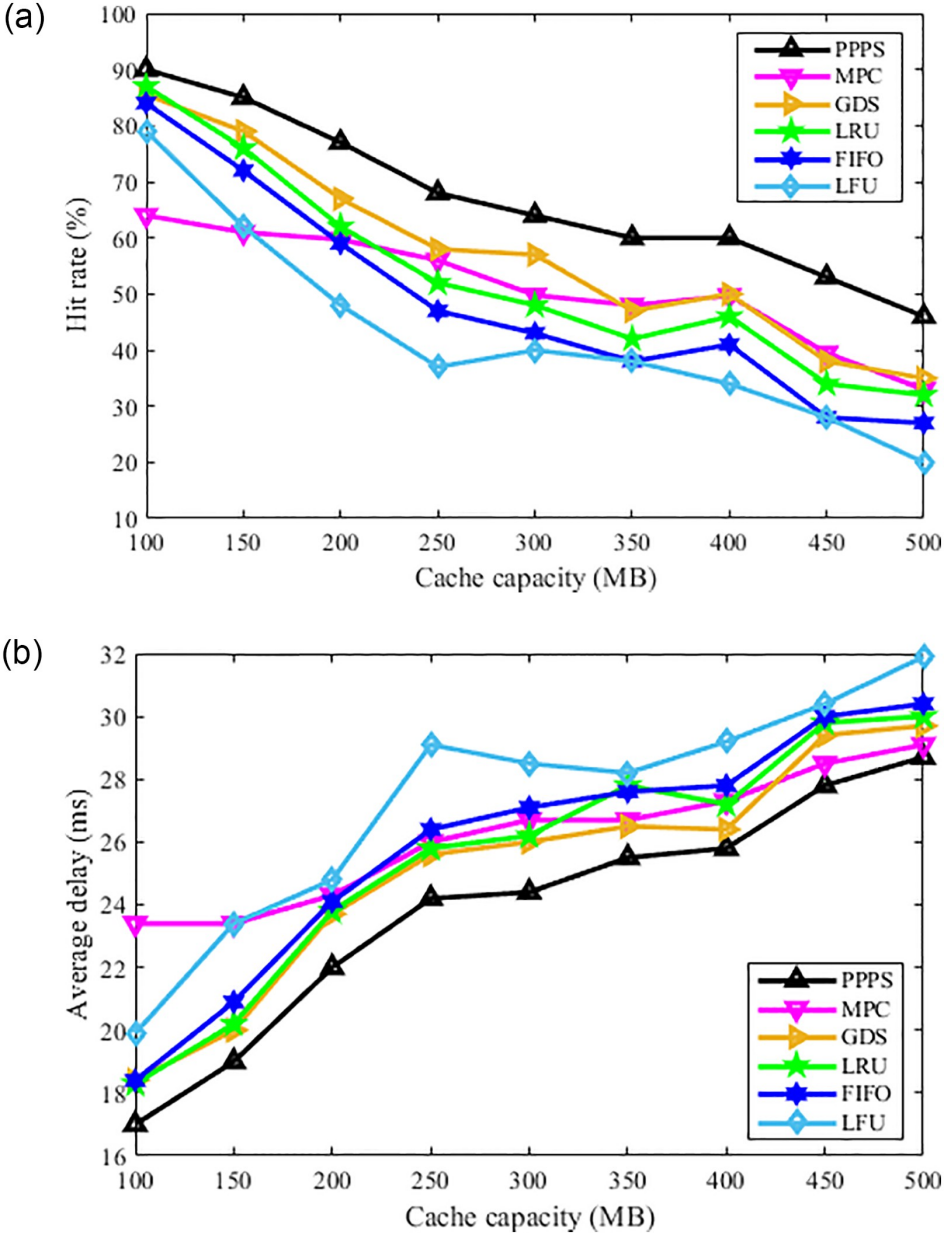
Comparison of the hit rate and average delay of the algorithms with different content types. (a) variation of hit rate with cache capacity. (b) variation of average delay with cache capacity.

2) Performance analysis of the caching algorithm when the content size is the same. The results of Experiment 2 correspond to Fig 6, which aims to explore the effect of the size of the content on the performance of the caching algorithm. Under the request traffic generated by the SNM model, the size of all content is set to be the same. Fig 6(a) shows the comparison of the hit rates of the replacement algorithms under different cache capacities. The performance of the MPC algorithm is the worst, because when the content size is set to 1 MB small files, the MPC sets a static popularity threshold, so even if the cache has sufficient space, a considerable part of the data cannot reach the popularity. The degree threshold cannot enter the cache. The LFU algorithm is second. The performance of LRU, GDS and FIFO is similar. The hit rate of the proposed algorithm remains the highest, and the performance improvement is more obvious as the cache capacity increases. When the cache capacity is 120 MB, the hit rate of the proposed algorithm is 5.5% higher than that of GDS, LRU and FIFO, 16.8% higher than LFU, and 20.7% higher than MPC. Fig 6(b) shows the comparison of the average delay of the algorithms, and the proposed algorithm has the smallest average delay. It can be seen that after removing the influencing factors of content size, the performance of the proposed algorithm is still the best, which confirms the effectiveness of the popularity prediction method based on attribute features.

3) Analysis of the influence of content types on algorithm performance. The results of Experiment 3 correspond to Fig 7, which aims to explore the performance of the caching algorithms under different content types. Under the request traffic generated by the SNM model, let the content types range from 100 to 500, Fig 7 shows the performance comparison of the algorithms. It can be seen that with the increase of content types, the hit rate of all cache algorithms gradually decreases, and the average delay increases gradually. Due to the problem of cache pollution, the performance of the LFU algorithm is low under various content types, and the average delay curve of the LFU algorithm has small fluctuations. This is because there are many small-sized content in the industry, and large-sized content with low request frequency is frequently replaced, and it is easy to lead to the increase of delay. The performance curves of the three algorithms, FIFO, LRU, and GDS, are similar, and they have different degrees of performance improvement on the basis of LFU. The performance of the MPC algorithm degrades when the cache capacity is large enough. As the content traffic increases, the performance of the MPC algorithm gradually outperforms the LRU algorithm. On the other hand, the proposed algorithm always maintains the highest hit rate and lower latency. When there are 350 types of content, the hit rate of proposed algorithm is 15.3% higher than GDS, 17.3% higher than MPC, 20.1% higher than LRU, 22.3% higher than FIFO, and 24.8% higher than LFU. Moreover, the performance improvement of the proposed algorithm compared with other algorithms increases with the increase of content types. In industrial applications, the diversity of content types may be faced, and the proposed algorithm maintains the best performance in different content type tests.

It can be seen from the experimental results of Fig 7, the content size, content type, and user request model all have an impact on the performance of the cache replacement algorithm, but the proposed algorithm always has the highest cache hit rate and lower average delay. It periodically predicts the popularity of feature attributes, incorporates content popularity prediction into the calculation of cache value, judge’s future hot content, and stores hot content in the edge cache in advance when the cache space is free, which effectively improves cache utilization and improves cache hit rate, and effectively reduce transmission delay.

**Fig 8:**
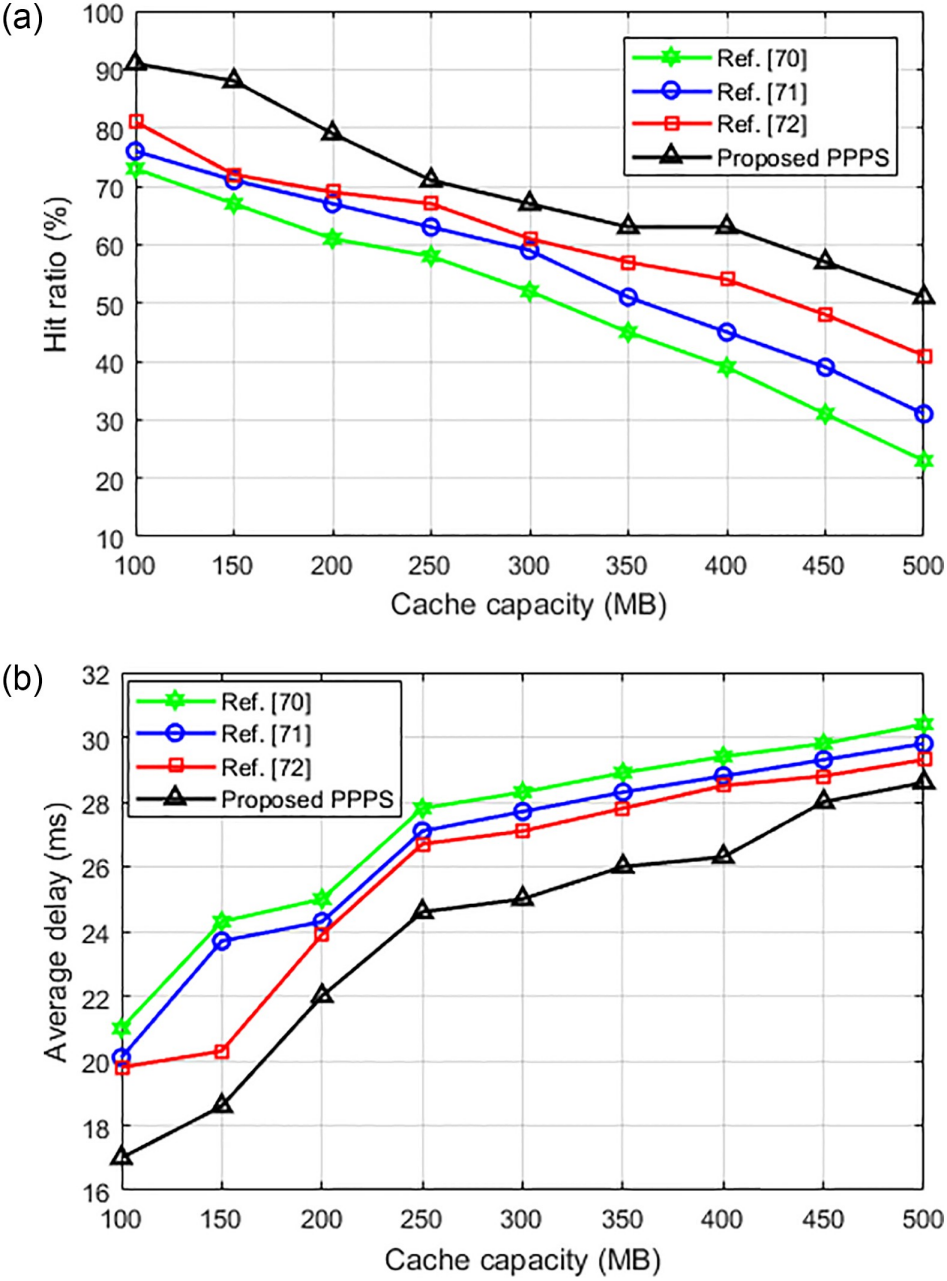
Comparison of the hit rate and average delay of the algorithms with dynamic contents. (a) variation of hit rate with cache capacity. (b) variation of average delay with cache capacity.

Fig 8 compared the hit ratio and latency delay of the proposed and references [28, 70, 71] algorithms under different cache resource capacity. As can be seen from Fig 8(a), the hit ratio of the proposed algorithm under dynamic contents is superior than that of existing algorithms which further validates its effectiveness. From Fig 8(b), we can see that the average delay of the proposed algorithm is lower than existing algorithms, which also proved its effectiveness.

**Fig 9:**
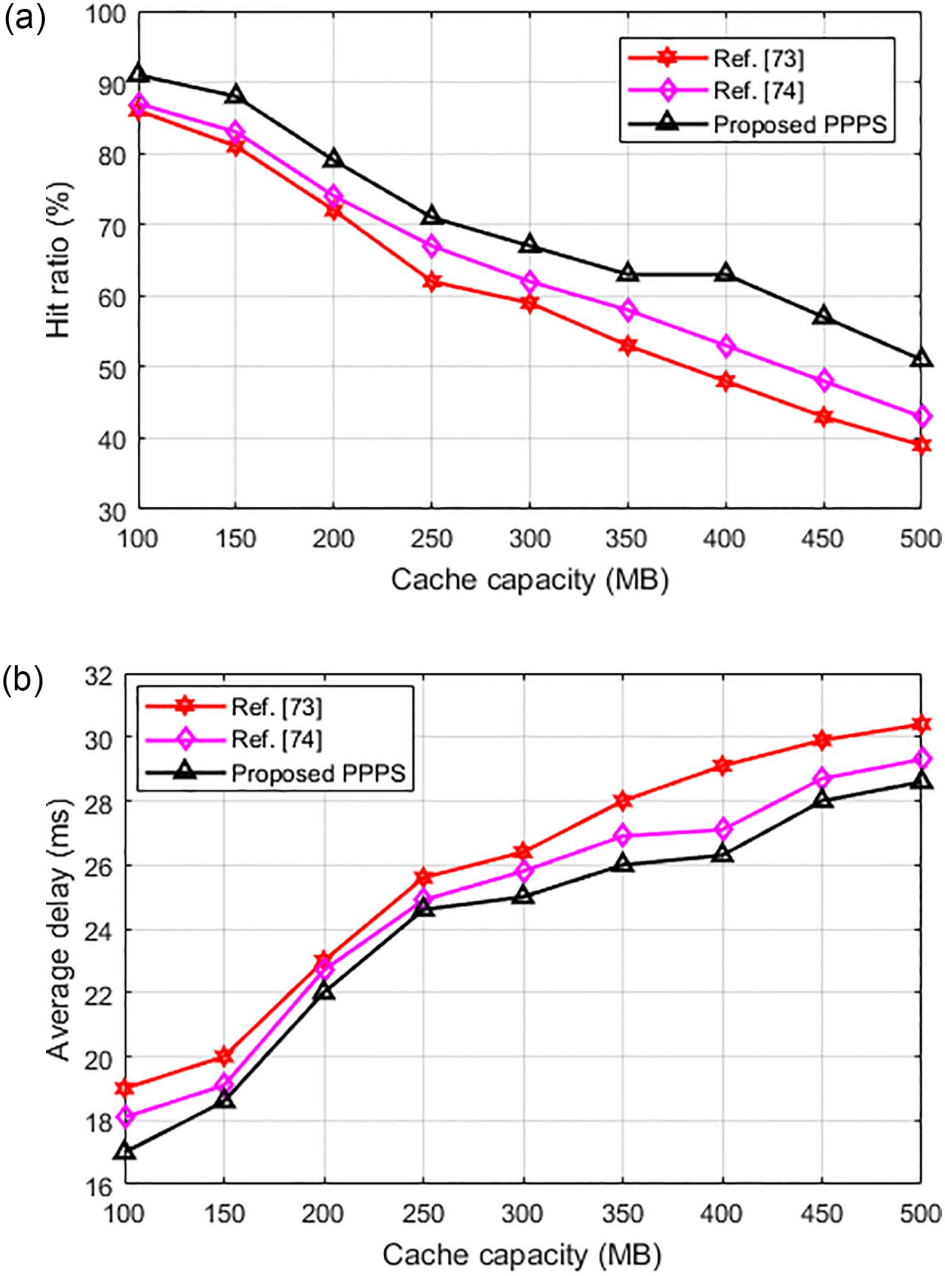
Performance comparison of proposed and existing algorithms. (a) variation of hit rate with cache capacity. (b) variation of average delay with cache capacity.

Fig 9 compare the hit ratio and average delay performance of the proposed and references [72, 73] algorithms. As can be seen that, the hit ratio of the proposed algorithm is better than existing algorithms. Also, the average delay of the proposed method is also lower than existing methods which further validated its effectiveness.

## 7 Limitations

The main limitations of this study are as follows:

- Performance degradation with increasing number of nodes.
- Unable to deploy in multi-dimensional features scenario.
- As the data popularity of blocks increases, the data response time and cache replacement number decreases.
- High data replacement rates.

## 8 Conclusion and future work

Based on the industrial edge computing application scenario, this paper first establishes an industrial edge network model and user request model. A novel single-dimensional content popularity probability model is proposed based on the feature changes of the content request sequence in the recent time window. It is implemented based on the content size cache replacement algorithm. After creating a shot noise model (SNM) model for industrial user requests, create a popularity change model for user requests. To ascertain the worth of the content, it carefully takes into account timeliness, content size priority, and popularity projection. The experimental results show that the proposed algorithm is comparable to five classical algorithms such as MPC, GDS, LRU, LFU and FIFO.

In comparison, under the two performance indicators of cache hit rate and average delay, the best performance is achieved under different content size distribution, content type, and user request model.

This work provides a basis for the selection of caching algorithms in practical industrial scenarios such as supply chain management, smart manufacturing, automation energy optimization, intelligent logistics transportation and es-healthcare applications.

The proposed algorithm has some shortcomings such as high data replacement rates, lower data response time with increasing blocks popularity, and lack of synchronization.

Future work will consider characterizing content popularity through multi-dimensional features and using machine learning algorithms to predict the changes in popularity cycles.
